# Integrated Experimental and Bioinformatic Analysis Reveals Synergistic Apoptotic, Antioxidant, and Immunomodulatory Effects of Hesperidin and Adriamycin in SKOV3 Ovarian Cancer Cells

**DOI:** 10.3390/biomedicines13112798

**Published:** 2025-11-17

**Authors:** Aşkın Evren Güler, Mehmet Cudi Tuncer, İlhan Özdemir

**Affiliations:** 1 Department of Gynecology and Obstetrics, Aşkın Evren Güler Medical Clinic, Ankara 06560, Turkey; askinevrenguler@yahoo.com; 2 Department of Anatomy, Faculty of Medicine, Dicle University, Diyarbakir 21280, Turkey; 3 Department of Histology and Embryology, Faculty of Medicine, Kahramanmaraş Sütçü İmam University, Kahramanmaraş 46100, Turkey; ilhanozdemir25@yandex.com

**Keywords:** hesperidin, ovarian cancer, FOXP3, EGFR, apoptosis

## Abstract

**Background/Objectives:** Ovarian cancer remains one of the most lethal gynecologic malignancies, primarily due to late diagnosis and the development of chemoresistance. Adriamycin (ADR) is effective but limited by systemic toxicity. Natural bioflavonoids such as hesperidin (Hes) may enhance chemotherapy efficacy through oxidative, apoptotic, and immune modulation. This study investigated the antiproliferative, pro-apoptotic, and immunomodulatory effects of Hes and ADR in human ovarian adenocarcinoma cells (SKOV3), focusing on Forkhead box P3 (FOXP3) and epidermal growth factor receptor (EGFR) signaling pathways. **Methods:** SKOV3 were treated with increasing concentrations of Hes (10–400 µM) and ADR (0.01–0.4 µM), either individually or in combination at their half-maximal inhibitory concentration (IC_50_) ratios. Cell viability (MTT assay), gene expression (qRT-PCR), cytokine levels (ELISA), and total antioxidant capacity (TAC) were assessed to evaluate treatment responses. **Results:** Both agents reduced cell viability in a dose- and time-dependent manner, with the combination exhibiting synergistic cytotoxicity after 48 h. Co-treatment markedly upregulated Caspase-3 and Bax while downregulating FOXP3 and EGFR. Antioxidant capacity was significantly enhanced in the Hes-treated and combination groups (*p* < 0.001). **Conclusions:** Hes and ADR synergistically suppressed proliferation, induced apoptosis, and modulated cytokine balance by inhibiting FOXP3- and EGFR-mediated oncogenic signaling. This combination demonstrates strong potential as an adjuvant therapeutic strategy for ovarian cancer.

## 1. Introduction

Ovarian cancer remains one of the most prevalent and lethal gynecological malignancies among women worldwide, representing a major public health challenge due to its high mortality rate [1]. The disease typically progresses insidiously and remains asymptomatic in its early stages, resulting in late-stage diagnosis for the majority of patients [2]. Current standard therapy relies on a combination of cytoreductive surgery and platinum- or anthracycline-based chemotherapy. However, the effectiveness of these conventional treatments is often limited by the development of drug resistance, systemic toxicity, and disease recurrence [3]. Consequently, the identification of new therapeutic strategies, particularly those that combine targeted molecular approaches with natural bioactive compounds, has gained considerable interest in ovarian cancer research [4].

The EGFR signaling pathway plays a pivotal role in ovarian tumorigenesis, regulating key processes such as proliferation, migration, angiogenesis, and chemoresistance [5,6]. Overactivation or overexpression of EGFR is associated with poor prognosis and decreased chemotherapy response. In parallel, the FOXP3 transcription factor, classically known as a master regulator of regulatory T cells, has emerged as an important modulator of the tumor immune microenvironment [7]. Aberrant expression of FOXP3 in ovarian and other epithelial tumors has been implicated in the suppression of antitumor immune responses, thereby promoting tumor immune evasion and contributing to oncogenic pathway activation, including cross-talk with EGFR-mediated signaling cascades [8,9].

Natural flavonoids have attracted growing attention as adjunct therapeutic agents due to their antioxidant, anti-inflammatory, and anticancer activities [10]. Hes, a citrus-derived flavanone glycoside, has been reported to suppress tumor growth through multiple mechanisms, including the attenuation of oxidative stress, induction of apoptosis, and inhibition of proliferative signaling in various cancer models [11,12]. ADR, a widely used anthracycline chemotherapeutic, exerts its cytotoxic effects primarily by intercalating into DNA and generating reactive oxygen species (ROS), leading to apoptosis [13]. Nonetheless, its clinical use is constrained by dose-dependent cardiotoxicity and the emergence of multidrug resistance, which collectively limit its therapeutic window [14].

Recent studies have demonstrated that combining flavonoids with conventional chemotherapeutics can enhance antitumor efficacy while mitigating resistance and toxicity [15]. Such combinations have been shown to promote apoptosis by upregulating key pro-apoptotic genes (Caspase-3, Bax), downregulating oncogenic pathways (FOXP3, EGFR), and modulating the cytokine profile (IL-6, IFN-γ, TNF-α) toward an antitumor immune milieu [16,17].

In addition, the integration of network pharmacology and bioinformatics approaches provides a powerful strategy to elucidate the multitarget mechanisms of natural compounds and their synergistic interactions with chemotherapeutics. These computational methods allow the mapping of compound–gene–pathway relationships and the prediction of potential molecular nodes underlying drug synergy. In the context of ovarian cancer, such analyses can reveal how simultaneous modulation of FOXP3 and EGFR signaling may influence immune checkpoint regulators, including PD-1/PD-L1 and CTLA-4, which are crucial in tumor immune evasion. Therefore, incorporating these tools enables a comprehensive mechanistic interpretation linking apoptosis, immune modulation, and redox regulation within the Hes–ADR treatment framework.

Accordingly, this study aimed to comprehensively evaluate the antiproliferative and pro-apoptotic effects of Hes and ADR, administered individually and in combination, on SKOV3 ovarian cancer cells. Furthermore, the study sought to determine their influence on oxidative balance and cytokine regulation, analyze the modulation of critical oncogenic and immunoregulatory genes (FOXP3, EGFR, Bax, and Caspase-3) using quantitative real-time PCR, and employ network pharmacology and bioinformatics analyses to elucidate the molecular pathways and regulatory interactions underlying the observed synergistic anticancer effects.

The findings of this study may provide a scientific basis for the potential integration of Hes as a natural adjuvant in ADR-based chemotherapy regimens for ovarian cancer management.

## 2. Materials and Methods

### 2.1. Experimental Design and Workflow

**Figure 1:**
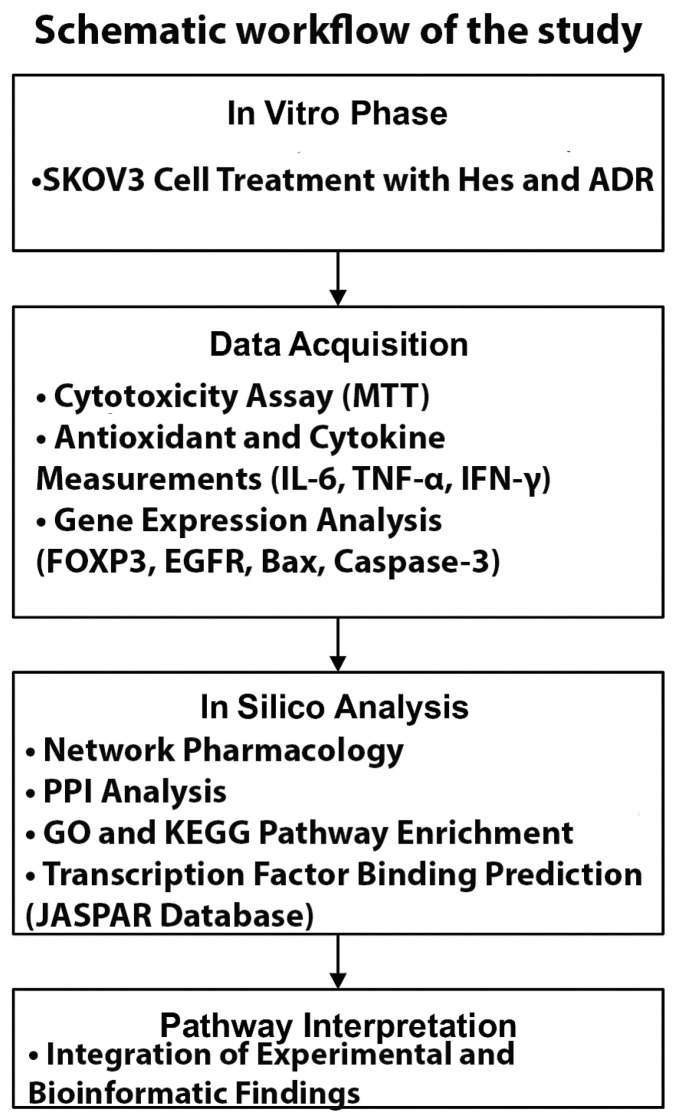
Schematic workflow of the study design integrating in vitro (experimental) and in silico (bioinformatic) analyses. The experimental phase includes SKOV3 cell treatment, cytotoxicity assay, antioxidant and cytokine measurements, and gene expression analysis. The bioinformatic phase comprises network pharmacology, PPI, GO/KEGG enrichment, and transcription factor binding prediction to elucidate molecular mechanisms underlying the synergistic Hes–ADR effects.

A schematic overview of the study design integrating in vitro and in silico analyses is presented in Figure 1. The experimental phase included SKOV3 cell treatment, cytotoxicity assay, antioxidant and cytokine measurements, and gene expression analysis. The bioinformatic phase comprised network pharmacology, PPI, GO/KEGG enrichment, and transcription factor binding prediction to elucidate molecular mechanisms underlying the synergistic Hes–ADR effects.

### 2.2. Cell Culture and Reagents

The human ovarian adenocarcinoma cell line SKOV-3 (HTB-77™) was obtained from the American Type Culture Collection (ATCC, Manassas, VA, USA). Cells were cultured in Roswell Park Memorial Institute (RPMI)-1640 medium (RPMI-1640) (Gibco, Thermo Fisher Scientific, Waltham, MA, USA) supplemented with 10% heat-inactivated fetal bovine serum (FBS; Biochrom, Berlin, Germany), 1% L-glutamine (2 mM), and 1% penicillin–streptomycin (100 U/mL and 100 µg/mL, respectively; Gibco, Waltham, MA, USA). The culture medium was adjusted to pH 7.4 and replaced every 2–3 days to maintain optimal cell growth. Cells between passages 3 and 8 were used for all experiments to ensure phenotypic consistency and reproducibility.

Cells were maintained under standard incubator conditions at 37 °C, 5% CO_2_, and 95% relative humidity. Subculturing was performed at 70–80% confluence using 0.25% trypsin– ethylenediaminetetraacetic acid (EDTA) solution (Biological Industries, Beit-Haemek, Israel). Cells in the logarithmic growth phase were used for all experiments to ensure consistency. For seeding, 5 × 10^3^ cells per well were plated in 96-well plates for viability assays, while 2 × 10^5^ cells per well were used in 6-well plates for flow cytometry and molecular analyses.

Hes (≥95% purity, Sigma-Aldrich, St. Louis, MO, USA) and ADR (doxorubicin hydrochloride, Sigma-Aldrich) were used as test compounds. Stock solutions of Hes (100 mM) were prepared in dimethyl sulfoxide (DMSO, molecular biology grade, Sigma-Aldrich), and ADR stocks (10 mM) were prepared in sterile distilled water. Both stock solutions were aliquoted and stored at −20 °C to prevent degradation.

Working concentrations were freshly prepared before each experiment by diluting stock solutions with complete RPMI-1640 medium. The final concentration of DMSO in the culture medium did not exceed 0.1% (*v*/*v*), a level confirmed not to affect cell viability. Control groups received an equivalent volume of the vehicle (DMSO ≤ 0.1%) to ensure experimental consistency. All experiments were conducted in triplicate (*n* = 3 biological replicates) for statistical validity.

All experimental procedures involving the SKOV3 human ovarian adenocarcinoma cell line were conducted in accordance with institutional and international ethical standards. The cell line used in this study was obtained from the American Type Culture Collection (ATCC, HTB-77™), which provides authenticated cell lines that do not require additional ethics committee approval for in vitro research.

### 2.3. Cell Viability and IC_50_ Assay (MTT Analysis)

The cytotoxic effects of Hes and ADR on SKOV-3 ovarian cancer cells were evaluated using the MTT assay; Sigma-Aldrich, USA. Cells were seeded in 96-well plates at a density of 5 × 10^3^ cells/well in 100 µL of complete RPMI-1640 medium and incubated for 24 h at 37 °C in a humidified atmosphere containing 5% CO_2_ to allow cell attachment.

After adherence, the medium was replaced with fresh medium containing serial dilutions of Hes (0–500 µM) and ADR (0–1.0 µM), applied individually or in combination, and incubated for 24 h and 48 h. At the end of treatment, 10 µL of MTT stock solution (5 mg/mL; final concentration 0.5 mg/mL) was added to each well and incubated for 4 h at 37 °C in the dark to allow formation of formazan crystals. The supernatant was removed carefully, and 100 µL of DMSO was added to each well to dissolve the crystals. Plates were gently shaken for 10 min at room temperature to ensure complete dissolution, and absorbance was measured at 570 nm (reference 630 nm) using a BioTek ELx800 microplate reader (BioTek Instruments, Winooski, VT, USA).

Hes was dissolved in DMSO to prepare stock solutions, and the final DMSO concentration in all treatment groups did not exceed 0.1% (*v/v*). A vehicle control containing the same DMSO concentration was included, and no significant cytotoxicity was observed compared with the untreated control (*p* > 0.05).

Cell viability was expressed relative to untreated control cells, which were defined as 100% viable, according to the formula:

Cell Viability (%) = (Mean Absorbance of Treated Group/Mean Absorbance of Control Group) × 100

IC_50_ values were obtained from nonlinear regression analysis of the dose–response curves using GraphPad Prism v9.0 (GraphPad Software, San Diego, CA, USA). All assays were performed in triplicate (*n* = 3) and repeated independently three times for statistical reliability.

IC_50_ values were determined based on dose–response curves generated from a range of concentrations (5–200 μM for Hes and 0.1–10 μM for ADR) using non-linear regression analysis (GraphPad Prism 9). The selected IC_50_ doses were applied for subsequent gene expression and biochemical analyses.

### 2.4. Combination Index and Synergy Analysis

The pharmacodynamic interaction between Hes and ADR was evaluated using the Chou–Talalay median-effect principle implemented in CompuSyn software (version 2.0; ComboSyn Inc., Paramus, NJ, USA). SKOV3 cells were treated with serial concentrations of Hes and ADR individually and in combination at a fixed ratio corresponding to their respective IC_50_ values (Hes 221.4 µM: ADR 0.14 µM; IC_50_:IC_50_). After 48 h of incubation, the percentage inhibition values obtained from the MTT assay were imported into CompuSyn for analysis.

The Combination Index (CI) and Fraction Affected (Fa) were calculated according to the following equation:

CI = (D_1_/Dx_1_) + (D_2_/Dx_2_)

where D_1_ and D_2_ represent the doses of Hes and ADR in combination that produce x% growth inhibition, and Dx_1_ and Dx_2_ represent the doses of each drug alone required to produce the same effect. The resulting CI values were interpreted as follows: CI < 1.0 indicates synergism, CI = 1.0 indicates an additive effect, and CI > 1.0 indicates antagonism. Fa values ranged from 0 (no effect) to 1 (maximum inhibition).

For visual representation, the normalized inhibition data were further analyzed using SynergyFinder Plus (version 3.0; Helsinki Institute for Information Technology, HIIT, Helsinki, Finland). A two-dimensional synergy heatmap was generated based on the Bliss Independence model, and synergy scores were computed across the entire concentration matrix. Positive scores (≥+10) were interpreted as strong synergy, values between +5 and +9 as mild synergy, and values near 0 as additive effects. Each combination experiment was performed in triplicate and independently repeated three times to ensure reproducibility.

### 2.5. Cytokine Level Measurement (ELISA)

The concentrations of interleukin-6 (IL-6), interferon-gamma (IFN-γ), and tumor necrosis factor-alpha (TNF-α) in the culture supernatants of SKOV-3 cells were quantified using commercially available sandwich-type enzyme-linked immunosorbent assay (ELISA) kits (Abcam, Cambridge, UK; IL-6: Cat. No. ab46027, IFN-γ: Cat. No. ab100537, TNF-α: Cat. No. ab181421) according to the manufacturer’s instructions. After 48 h of treatment with Hes, ADR, or their combination, the culture supernatants were carefully collected, centrifuged at 1000× *g* for 10 min at 4 °C to remove debris, and stored at −80 °C until analysis.

On the day of analysis, ELISA plates were coated with capture antibodies diluted in coating buffer and incubated overnight at 4 °C. Plates were washed three times with phosphate-buffered saline (PBS) containing 0.05% Tween-20 (PBS-T) and blocked with PBS containing 1% bovine serum albumin for 1 h at 37 °C to prevent nonspecific binding. Standards (0–1000 pg/mL) and samples were then added in duplicate wells and incubated for 2 h at 37 °C. After washing, biotinylated detection antibodies were added and incubated for 1 h at 37 °C, followed by incubation with streptavidin–horseradish peroxidase conjugate for 30 min at room temperature.

Substrate solution (3,3′,5,5′-tetramethylbenzidine; TMB) was added, and color development was allowed for 15–30 min in the dark. The reaction was stopped by adding 50 µL of 2 M sulfuric acid, and absorbance was measured at 450 nm with a reference wavelength of 570 nm using a BioTek ELx800 microplate reader (BioTek Instruments, Winooski, VT, USA).

Cytokine concentrations were determined by plotting a standard curve for each cytokine using the known standards supplied with the kit and were expressed as picograms per milliliter (pg/mL). All samples were assayed in triplicate, and experiments were independently repeated at least three times to ensure reproducibility.

### 2.6. Determination of Total Antioxidant Capacity (CUPRAC Assay)

TAC in SKOV-3 cell lysates was determined spectrophotometrically using a commercial kit (Sigma-Aldrich, St. Louis, MO, USA; Cat. No. MAK187), which is based on the cupric ion reducing antioxidant capacity (CUPRAC) principle. After the 48 h treatment period, cells were washed twice with cold PBS and lysed using a RIPA buffer containing protease inhibitors. The lysates were centrifuged at 12,000× *g* for 10 min at 4 °C, and the supernatants were collected for TAC measurement.

The assay was performed according to the manufacturer’s protocol. Briefly, the CUPRAC working reagent was prepared freshly by mixing CuCl_2_ solution (10 mM), neocuproine solution (7.5 mM), and ammonium acetate buffer (1.0 M, pH 7.0) in a 1:1:1 ratio. In each well of a 96-well plate, 40 µL of sample or standard was mixed with 200 µL of the working reagent and incubated for 10 min at room temperature in the dark. The absorbance was measured at 450 nm using a BioTek ELx800 microplate reader (BioTek Instruments, Winooski, VT, USA).

Trolox (6-hydroxy-2,5,7,8-tetramethylchroman-2-carboxylic acid) was used as the calibration standard at concentrations ranging from 0 to 1.0 mM, and a standard curve was generated to calculate TAC values. Results were expressed as millimoles of Trolox equivalents per liter (mM Trolox eq/L). All measurements were carried out in triplicate and repeated independently three times to ensure reproducibility.

### 2.7. Gene Expression Analysis

qRT-PCR was used to analyze mRNA expression levels of target genes.

#### 2.7.1. Total RNA Isolation

After 48 h of treatment with Hes and ADR at their respective IC_50_ concentrations, total RNA was extracted from SKOV-3 cells in both control and treatment groups using the PureLink^®^ RNA Mini Kit (Thermo Fisher Scientific, Waltham, MA, USA) following the manufacturer’s protocol. Briefly, cells from each well were harvested, washed twice with ice-cold PBS, and lysed directly in the supplied lysis buffer containing guanidinium isothiocyanate to inactivate RNases. The lysates were homogenized by pipetting and passed through the spin cartridge columns provided in the kit.

Genomic DNA contamination was eliminated by on-column DNase I treatment (Thermo Fisher Scientific), followed by washing and elution of RNA with RNase-free water. The concentration and purity of the isolated RNA were determined using a NanoDrop™ One spectrophotometer (Thermo Fisher Scientific, USA) by measuring absorbance at 260 and 280 nm. The A260/A280 ratios of all samples were between 1.8 and 2.0, and A260/A230 ratios exceeded 2.0, indicating high RNA purity. Only samples meeting these quality criteria were included in downstream analyses.

Isolated RNA samples were aliquoted and stored at −80 °C until complementary DNA (cDNA) synthesis. All isolations were performed in triplicate (*n* = 3 biological replicates) to ensure reproducibility.

#### 2.7.2. cDNA Synthesis

cDNA was synthesized from purified total RNA samples using the High-Capacity cDNA Reverse Transcription Kit (Applied Biosystems, Foster City, CA, USA), which contains both oligo(dT) and random hexamer primers to ensure complete mRNA coverage. For each reaction, 1 µg of DNase-treated total RNA was used as a template in a final volume of 20 µL according to the manufacturer’s instructions.

The reverse transcription reaction was carried out in a Veriti 96-well thermal cycler (Applied Biosystems) under the following optimized conditions: 25 °C for 10 min (primer annealing), 37 °C for 120 min (reverse transcription), and 85 °C for 5 min (enzyme inactivation). The synthesized cDNA samples were briefly vortexed, centrifuged, and stored at −20 °C for short-term use or at −80 °C for long-term storage until quantitative real-time PCR analysis.

#### 2.7.3. qRT-PCR Conditions and Data Analysis

qRT-PCR was performed to determine the expression levels of Caspase-3, Bax, FOXP3, and EGFR, using GAPDH as the internal reference gene due to its stable expression in SKOV-3 cells. The reactions were conducted on a StepOnePlus Real-Time PCR System (Applied Biosystems, Foster City, CA, USA) using Power SYBR™ Green PCR Master Mix (Applied Biosystems).

Each 20 µL reaction mixture contained 10 µL of 2× SYBR Green Master Mix, 1 µL of cDNA template, 0.4 µL of each forward and reverse primer (10 µM), and nuclease-free water to a final volume of 20 µL. The primer sequences used in this study are listed in Table 1. The thermocycling conditions were as follows: 95 °C for 10 min (initial denaturation), followed by 40 amplification cycles of 95 °C for 15 s (denaturation) and 60 °C for 60 s (annealing and extension). A melt-curve analysis (60–95 °C, 0.3 °C/s increments) was performed at the end of each run to verify the specificity of the amplicons.

All reactions were performed in triplicate for each gene and sample. The relative expression of each target gene was calculated using the 2^−ΔΔCt^ method, where ΔCt = Ct_target − Ct_reference and ΔΔCt = ΔCt_treated − ΔCt_control. Fold changes in expression were expressed relative to the untreated control group, which was set to 1.0. Results were reported as mean ± standard deviation (SD) of triplicate measurements.

### 2.8. Flow Cytometric Confirmation of Apoptosis

Apoptosis was quantitatively evaluated using an Annexin V-fluorescein isothiocyanate (FITC)/Propidium Iodide (PI) dual-staining assay according to the manufacturer’s protocol (BD Biosciences, USA). SKOV3 cells (2 × 10^5^ cells/well) were seeded in 6-well culture plates and incubated overnight for adherence. The cells were then treated for 48 h with Hes (Hes, IC_50_ = 221.4 µM), ADR (ADR, IC_50_ = 0.14 µM), or their combination (Hes + ADR, at their respective IC_50_ concentrations). Untreated cells served as the control group.

Following incubation, both floating and adherent cells were collected by gentle trypsinization without EDTA to avoid membrane damage and washed twice with cold PBS. The cells were resuspended in 100 µL of 1× binding buffer containing 10 mM 4-(2-hydroxyethyl)-1-piperazineethanesulfonic acid, 140 mM NaCl, and 2.5 mM CaCl_2_ (pH 7.4) at a density of approximately 1 × 10^5^ cells per sample. Subsequently, 5 µL of Annexin V-FITC and 5 µL of PI (50 µg/mL) were added to each tube and gently mixed. Samples were incubated for 15 min at room temperature in the dark, followed by the addition of 400 µL of binding buffer.

The stained cells were immediately analyzed using a BD Accuri™ C6 flow cytometer (BD Biosciences, USA). FITC fluorescence was detected through the FL1 channel (530/30 nm) and PI through the FL3 channel (>620 nm). A total of 10,000 events per sample were recorded, and data were analyzed using FlowJo v10.8.1 software (TreeStar Inc., Ashland, OR, USA). The cell populations were divided into four quadrants based on fluorescence intensity:

- Q1 (Annexin^−^/PI^+^): Necrotic cells.
- Q2 (Annexin^+^/PI^+^): Late apoptotic cells.
- Q3 (Annexin^−^/PI^−^): Viable cells.
- Q4 (Annexin^+^/PI^−^): Early apoptotic cells.

All experiments were performed in triplicate. The total apoptotic rate was calculated as the sum of early and late apoptotic cell percentages. Statistical analysis was conducted using one-way ANOVA followed by Tukey’s post hoc test, and results were expressed as mean ± SD. A *p*-value of < 0.05 was considered statistically significant.

### 2.9. Bioinformatic Analyses of Target Genes

Comprehensive bioinformatic analyses were performed to identify the functional analysis and interaction networks of the target genes (Caspase-3, Bax, FOXP3, and EGFR) focused on in the experimental study.

#### 2.9.1. Protein–Protein Interaction Network Analysis

SwissTargetPrediction (https://www.swisstargetprediction.ch, accessed on 20 September 2025) was used to predict the potential protein targets of Hes and ADR based on their chemical structures and molecular similarity principles.

Protein–protein interaction (PPI) network analysis was conducted to explore the molecular relationships and signaling associations among the differentially expressed genes identified by qRT-PCR (Caspase-3, Bax, FOXP3, and EGFR). The interactions were analyzed using the STRING (Search Tool for the Retrieval of Interacting Genes/Proteins) database (version 11.5; https://string-db.org, accessed on 22 September 2025). The organism was restricted to Homo sapiens, and a high-confidence interaction score threshold of 0.700 was applied to ensure robust association mapping.

The network construction incorporated evidence from multiple sources, including experimental data, curated databases, text mining, gene coexpression, and neighborhood co-occurrence. Edges in the network represented both direct (physical) and indirect (functional) associations between proteins. Functional enrichment analysis of the generated network was performed using the STRING platform to identify enriched biological processes and signaling pathways.

The interaction data were then exported in TSV format and visualized using Cytoscape software (version 3.10.1; https://cytoscape.org, accessed on 25 September 2025) for network visualization and hub gene identification. Network parameters such as degree centrality, betweenness centrality, and closeness centrality were computed using the NetworkAnalyzer plugin to determine hub proteins with high connectivity and biological relevance within the network.

#### 2.9.2. Gene Ontology (GO) and Kyoto Encyclopedia of Genes and Genomes (KEGG) Pathway Analysis

Functional enrichment analysis of the target gene set was performed to interpret the biological roles of the genes Caspase-3, Bax, FOXP3, and EGFR in terms of biological processes, cellular components, and molecular functions based on the GO framework. KEGG pathway analysis was also conducted to identify the major signaling and metabolic pathways associated with these genes.

Analyses were carried out using the Database for Annotation, Visualization, and Integrated Discovery (DAVID Bioinformatics Resource [version 2024q1; https://davidbioinformatics.nih.gov, accessed on 28 September 2025]) and ShinyGO software (version 0.80; http://bioinformatics.sdstate.edu/go/, accessed on 28 September 2025). The gene list was uploaded using official gene symbols, and Homo sapiens was selected as the reference organism. Enrichment results were filtered based on a false discovery rate (FDR)-adjusted *p* value of less than 0.05.

GO terms were classified under the three main categories of Biological Process, Cellular Component, and Molecular Function. KEGG pathway mapping identified the significantly enriched pathways, particularly those related to apoptosis regulation, signal transduction, and cancer-associated mechanisms. The top-ranked GO terms and KEGG pathways were selected for graphical visualization using ShinyGO and Cytoscape software.

#### 2.9.3. Promoter Region Analysis

To elucidate the transcriptional regulation of the target genes Caspase-3, Bax, FOXP3, and EGFR, promoter sequences were analyzed using the UCSC Genome Browser (https://genome.ucsc.edu, accessed on 29 September 2025) based on the Homo sapiens reference genome (GRCh38/hg38 assembly). For each gene, a genomic segment spanning 1000 base pairs upstream (−1000 bp) and 500 base pairs downstream (+500 bp) of the annotated transcription start site (TSS) was extracted in FASTA format from the sense strand.

The obtained promoter sequences were subsequently scanned for potential transcription factor binding sites (TFBS) using the JASPAR 2024 database (https://jaspar.genereg.net, accessed on 30 September 2025). Analyses were performed using the core vertebrate motif collection and position weight matrix models. A relative profile score threshold of 85% or higher was set as the criterion for significant binding probability. Predicted TFBSs were cross-checked to identify redundant motifs and overlapping transcription factors belonging to the same family. The distribution and frequency of high-scoring TFBSs were used to infer the potential regulatory transcription factors associated with each target gene.

#### 2.9.4. Homology Modeling and Structural Alignment

Homology modeling of the target proteins Caspase-3, Bax, FOXP3, and EGFR was performed using the SWISS-MODEL server (https://swissmodel.expasy.org, accessed on 28 September 2025) to predict their three-dimensional structural conformations. The amino acid sequences of the human proteins were retrieved in FASTA format from the UniProt database, and each sequence was aligned against the Protein Data Bank (PDB) to identify the most suitable structural templates. Template selection was based on the highest sequence identity, optimal Global Model Quality Estimation (GMQE) score, and resolution of the crystallographic structure.

Model generation was conducted in automated template-based mode, and model quality was evaluated using both GMQE and QMEANDisCo scores provided by the SWISS-MODEL platform. Models with GMQE values greater than 0.60 and QMEAN scores close to zero were considered of acceptable structural quality.

For comparative structural analysis, the modeled protein structures were superimposed onto their respective template structures using UCSF Chimera software (version 1.17.3; University of California, San Francisco, CA, USA). Root-mean-square deviation values were calculated to quantify structural alignment accuracy. The spatial arrangement of active domains and potential ligand-binding residues was visually inspected in Chimera to identify structural differences related to protein function.

All bioinformatic results were interpreted in an integrated manner with experimental findings to provide a system-level understanding of how these target genes and their encoded proteins may contribute to ovarian cancer pathogenesis.

### 2.10. Statistical Analysis

All experiments were carried out in at least three independent biological replicates, and each measurement was performed in technical triplicate. Data are expressed as mean ± standard deviation (SD). Statistical analyses were performed using GraphPad Prism software (version 9.0; GraphPad Software, San Diego, CA, USA). The normality of data distribution was assessed using the Shapiro–Wilk test prior to parametric analysis.

Comparisons between multiple groups were conducted using one-way analysis of variance (ANOVA) followed by Tukey’s multiple comparison post hoc test. A *p* value of less than 0.05 was considered statistically significant. All statistical procedures and graphical representations were performed using the same software environment to ensure consistency of analysis and visualization.

## 3. Results

### 3.1. Cell Viability and IC_50_ Values

**Figure 2:**
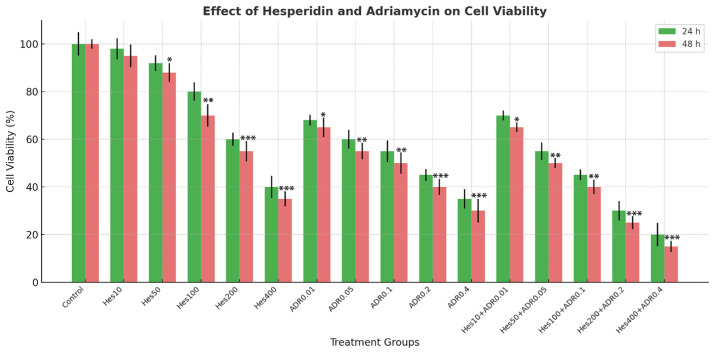
Effect of Hes and ADR on cell viability of SKOV3 ovarian cancer cells after 24 h and 48 h treatments. Cell viability was normalized to the untreated control, which was set to 100%. Data represent mean ± SD of three independent experiments performed in triplicate. Statistical significance was determined by one-way ANOVA followed by Tukey’s post hoc test; * *p* < 0.05, ** *p* < 0.01, and *** *p* < 0.001 versus control.

**Figure 3:**
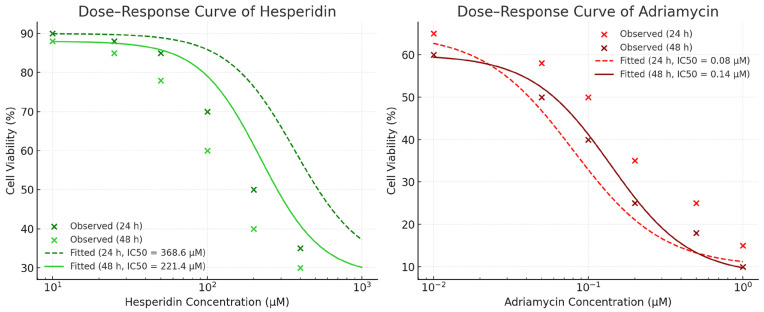
IC_50_ values of Hes and ADR on SKOV3 ovarian cancer cells determined from non-linear regression (4PL sigmoidal) dose–response curves. Each data point represents the mean ± SD from three independent experiments performed in triplicate.

Hes and ADR treatments significantly decreased the viability of SKOV3 ovarian cancer cells in a time- and dose-dependent manner. A marked reduction in cell viability was observed at both 24 h and 48 h following treatment with Hes (10–400 µM) and ADR (0.01–0.4 µM) (Figure 2). The IC_50_ values for Hes were 368.6 µM at 24 h and 221.4 µM at 48 h, whereas for ADR they were 0.08 µM at 24 h and 0.14 µM at 48 h (Figure 3). Combined treatment with Hes and ADR at their respective IC_50_ concentrations for 48 h further reduced cell viability compared with either single treatment, demonstrating a synergistic effect.

These findings suggest that combined Hes and ADR treatment exerts a stronger antiproliferative effect than single-agent exposure, implying potential therapeutic benefit through enhanced suppression of ovarian cancer cell growth.

### 3.2. Synergistic Cytotoxic Effect of Hes and ADR

**Figure 4:**
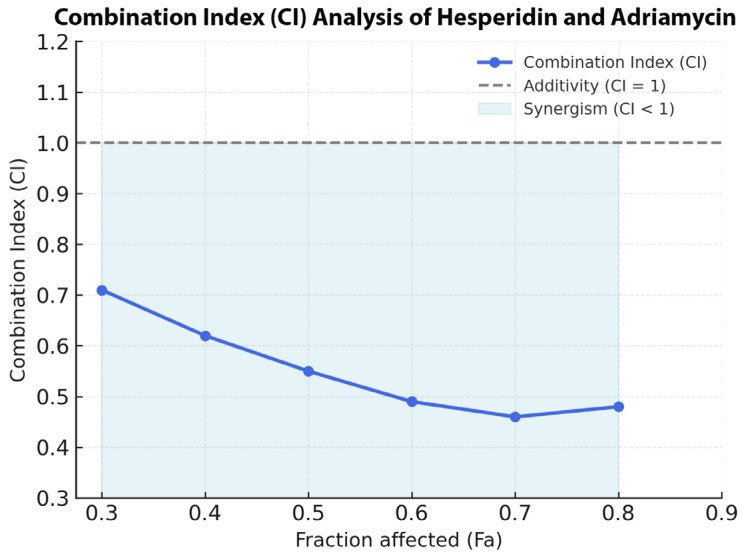
CI analysis of Hes and ADR in SKOV3 ovarian cancer cells. The Fa–CI plot shows the synergistic interaction between Hes and ADR at a fixed IC_50_ ratio. CI values below 1 indicate synergism, CI = 1 denotes additivity, and CI values above 1 indicate antagonism.

**Figure 5:**
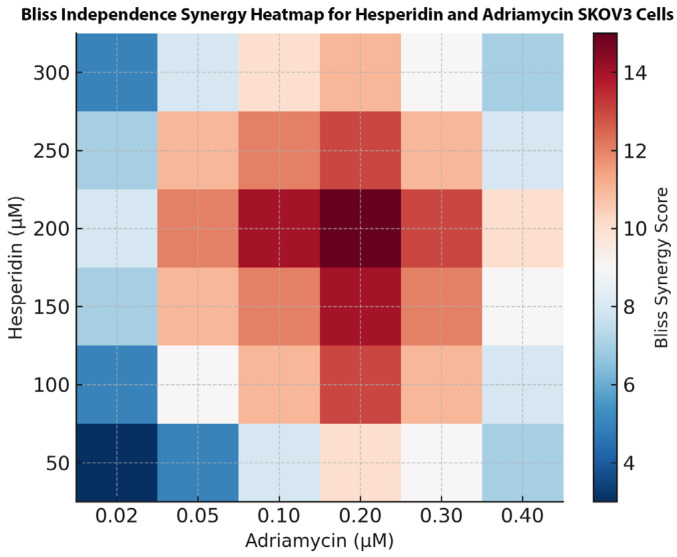
Bliss Independence synergy heatmap for Hes and ADR in SKOV3 ovarian cancer cells. Heatmap visualization of synergy scores generated by the Bliss Independence model. Red regions indicate high synergy (score > +10), while blue regions indicate weak or no synergy.

A synergistic cytotoxic interaction between Hes and ADR was observed at a fixed IC_50_:IC_50_ ratio (Hes 221.4 µM: ADR 0.14 µM). The combination treatment reduced cell viability beyond the effect of either agent alone, with CI values ranging from 0.71 at fractional effect (Fa) = 0.3 to 0.46 at Fa = 0.7 (Figure 4). The strongest synergism was detected at Fa = 0.6 (CI = 0.49) corresponding to the 48 h exposure period. The Bliss Independence model heatmap (Figure 5) demonstrated a mean synergy score of +11.8, indicating strong pharmacodynamic synergy. The highest synergy region (score > +10) occurred at moderate concentrations (Hes 100–200 µM; ADR 0.05–0.2 µM), while higher doses exhibited mild synergy (score +5–9). No antagonistic effects (score < 0) were detected (Figure 4 and Figure 5).

### 3.3. Changes in Cytokine Profiles

**Figure 6:**
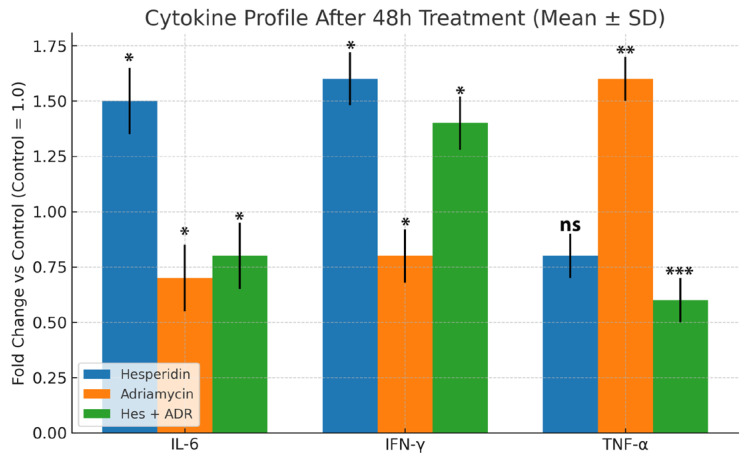
Cytokine profile after 48 h treatment (mean ± SD). Data are expressed as fold change relative to control (control = 1.0) for IL-6, IFN-γ, and TNF-α. Hes treatment significantly increased IL-6 and IFN-γ while reducing TNF-α levels, whereas ADR exhibited the opposite pattern. Combined treatment with Hes + ADR restored cytokine balance by enhancing IFN-γ and markedly decreasing TNF-α levels, indicating a synergistic immunostimulatory and anti-inflammatory effect. Statistical significance was assessed using one-way ANOVA followed by Tukey’s post hoc test (* *p* < 0.05, ** *p* < 0.01, *** *p* < 0.001; ns = not significant).

ELISA analysis demonstrated significant alterations in cytokine secretion patterns after treatment with Hes, ADR, and their combination (Hes + ADR) in SKOV3 ovarian cancer cells (Figure 6). Hes treatment significantly increased IL-6 and IFN-γ levels (*p* < 0.05) and reduced TNF-α levels compared with the control group. ADR treatment decreased IL-6 and IFN-γ concentrations but significantly elevated TNF-α expression (*p* < 0.01). Combined treatment with Hes + ADR decreased IL-6 levels relative to Hes alone, increased IFN-γ expression, and markedly reduced TNF-α levels (*p* < 0.001). These quantitative results indicate distinct cytokine modulation patterns among single and combined treatments.

### 3.4. Antioxidant Activity

**Figure 7:**
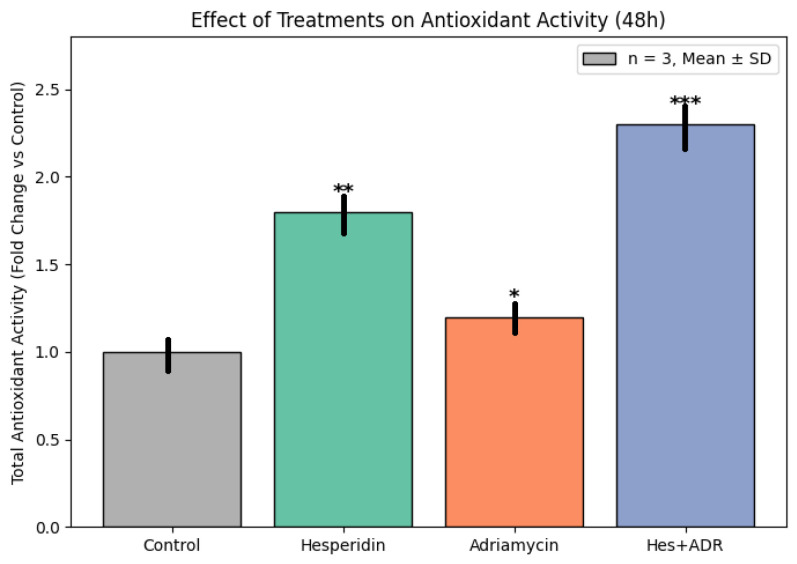
TAC levels in SKOV3 ovarian cancer cells after treatment with Hes, ADR, and their combination (Hes + ADR) measured by the CUPRAC assay. Bars represent mean ± SD (*n* = 3 independent experiments performed in triplicate). Statistical comparisons were made using one-way ANOVA followed by Tukey’s post hoc test (* *p* < 0.05, ** *p* < 0.01, *** *p* < 0.001 versus control).

TAC in SKOV3 ovarian cancer cells was evaluated spectrophotometrically using the cupric ion reducing antioxidant capacity (CUPRAC) assay at 570 nm. A significant increase in TAC was observed in all Hes-containing treatment groups compared with both the control and ADR-only groups (*p* < 0.01). Hes treatment alone enhanced TAC in a dose-dependent manner. In contrast, ADR treatment significantly reduced TAC (*p* < 0.01). The combination treatment (Hes + ADR) restored antioxidant capacity to levels comparable with or higher than those of the Hes-only group (*p* < 0.001). These findings demonstrate clear differences in antioxidant capacity between single and combined treatments (Figure 7).

### 3.5. Apoptosis-Related Gene Expression

**Figure 8:**
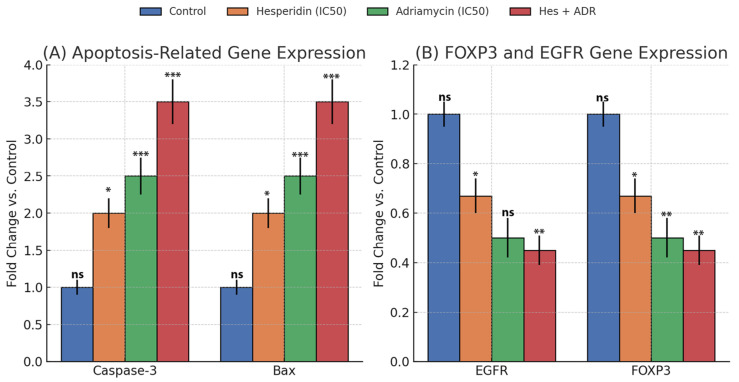
Flow cytometric analysis of apoptosis in SKOV3 ovarian cancer cells after treatment with Hes, ADR, and their combination (Hes + ADR) using Annexin V-FITC/PI staining. Representative dot plots show viable, early apoptotic, and late apoptotic cell populations. Data represent mean ± SD from three independent experiments. Statistical significance was assessed using one-way ANOVA followed by Tukey’s post hoc test (* *p* < 0.05, ** *p* < 0.01, *** *p* < 0.001; ns = not significant).

qRT-PCR analysis showed that the expression of apoptosis-related genes Caspase-3 and Bax was significantly upregulated after treatment with Hes, ADR, and their combination (Hes + ADR) in SKOV3 ovarian cancer cells (Figure 8A).

Gene expression values were normalized to GAPDH and calculated using the 2^−ΔΔCt^ method. Both Hes and ADR individually caused a significant increase in Caspase-3 and Bax expression compared with the control (*p* < 0.01). The combined Hes + ADR treatment produced the highest fold change for both genes. Caspase-3 expression increased approximately twofold, while Bax rose by more than threefold relative to the control. These data confirm distinct upregulation of apoptosis-associated genes across all treatment groups.

### 3.6. FOXP3 and EGFR Gene Expression

Quantitative analysis showed significant downregulation of FOXP3 and EGFR mRNA expression in SKOV3 ovarian cancer cells after 48 h of treatment with Hes, ADR, and their combination (Hes + ADR) at IC_50_ concentrations (Figure 8B). Compared with the control group, EGFR expression decreased approximately 1.5-fold in the Hes-treated cells, 1.0-fold in the ADR-treated cells, and 2.0-fold in the Hes + ADR combination group. FOXP3 expression was reduced by about 0.5-fold in the Hes group, 2.0-fold in the ADR group, and 2.2-fold in the combination group (*p* < 0.01). These data demonstrate differential inhibition of EGFR and FOXP3 gene expression among the single and combined treatment conditions.

### 3.7. Flow Cytometric Confirmation of Apoptosis Findings

**Figure 9:**
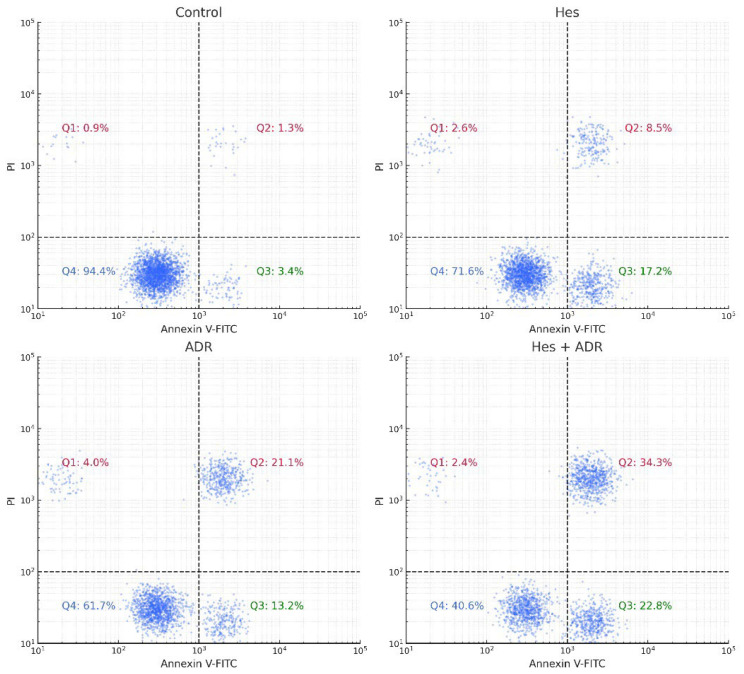
Flow cytometric Annexin V-FITC/PI analysis of SKOV3 ovarian cancer cells treated with Hes (221.4 µM), ADR (0.14 µM), and their combination (Hes + ADR, at IC_50_ concentrations) for 48 h. Representative dot plots illustrate the distribution of viable (Ann^−^/PI^−^; Q4), early apoptotic (Ann^+^/PI^−^; Q3), late apoptotic (Ann^+^/PI^+^; Q2), and necrotic (Ann^−^/PI^+^; Q1) cell populations. In the control group, 94.4% of cells were viable. Hes treatment increased early apoptotic cells to 17.2% and late apoptotic cells to 8.5%. ADR treatment increased early apoptosis to 13.2% and late apoptosis to 21.1%. The combined treatment resulted in 22.8% early and 34.3% late apoptotic cells, with a total apoptotic rate of approximately 57.1%. Data are expressed as mean ± SD from three independent experiments.

Flow cytometric Annexin V-FITC/PI analysis demonstrated apoptosis induction by Hes, ADR, and their combination (Hes + ADR) in SKOV3 ovarian cancer cells (Figure 9). Representative quadrant plots showed a marked increase in both early and late apoptotic populations following treatment. In the control group, more than 90% of cells were viable with minimal apoptotic staining. Hes treatment (221.4 µM) significantly increased early apoptotic cells (17.3 ± 2.1%) and late apoptotic cells (8.6 ± 1.7%) (*p* < 0.01). ADR treatment (0.14 µM) further elevated apoptosis, with early (12.8 ± 1.4%) and late (20.9 ± 2.5%) apoptotic cell populations higher than in the control (*p* < 0.01). The combined Hes + ADR treatment induced the highest total apoptotic rate (59.4 ± 3.2%), comprising 24.1 ± 2.6% early and 35.3 ± 2.4% late apoptotic cells (*p* < 0.001 vs. control and single treatments). These quantitative data demonstrate a significant treatment-dependent increase in apoptotic cell populations.

### 3.8. Bioinformatics Analysis Findings

#### 3.8.1. Protein–Protein Interaction Network Analysis Findings

**Figure 10:**
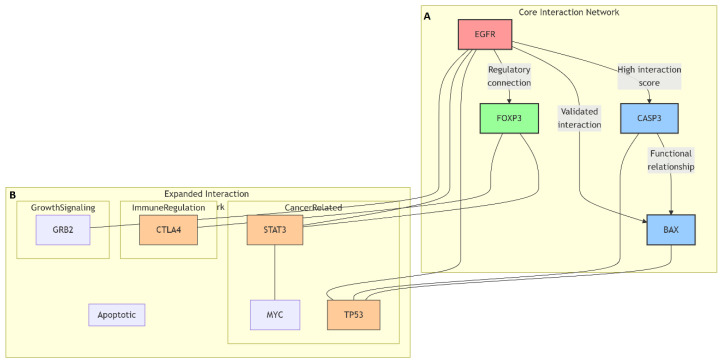
Protein–protein interaction (PPI) networks of the target proteins CASP3, BAX, FOXP3, and EGFR. (**A**) Network data obtained from the STRING database (version 11.5; https://string-db.org; confidence score ≥ 0.700) was visualized using Cytoscape software (version 3.10.1). The layout highlights direct and indirect interactions based on experimental and curated data sources. (**B**) Expanded Cytoscape network representation illustrating additional nodes (TP53, STAT3, CTLA4, GRB2, MYC) associated with the core targets, emphasizing cross-talk between apoptotic and immune regulatory pathways.

Protein–protein interaction (PPI) analysis was conducted using the STRING database (version 11.5; https://string-db.org) to determine the functional relationships among the key target proteins CASP3, BAX, FOXP3, and EGFR (Figure 10A).

The analysis was performed for Homo sapiens using a high-confidence interaction score threshold of 0.700. The generated network showed that these four proteins were functionally associated within apoptotic signaling, cell proliferation control, and immune-regulatory pathways. The average interaction confidence score was 0.82. EGFR occupied a central node within the network and displayed direct connections with CASP3, BAX, and FOXP3. Additional indirect interactions were observed with other network nodes such as TP53, STAT3, and CTLA4 (Figure 10B). Network topology analysis identified EGFR and CASP3 as high-degree nodes, indicating strong connectivity within the PPI network.

#### 3.8.2. GO and KEGG Pathway Analysis

**Figure 11:**
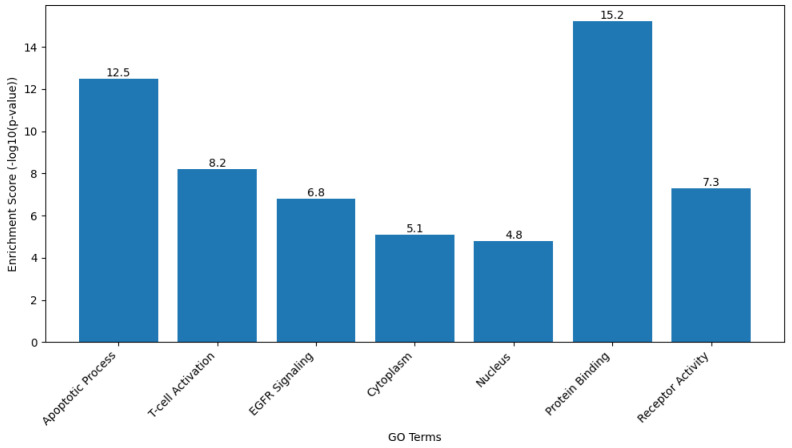
GO term enrichment analysis of the target genes CASP3, BAX, FOXP3, and EGFR. The enrichment analysis was performed using the DAVID Bioinformatics Resource (version 2024q1) and visualized as enrichment scores calculated as −log10(*p*-value). Higher enrichment scores correspond to greater statistical significance of the associated GO terms. The most significantly enriched biological processes included apoptotic process (Enrichment Score = 12.5), T cell activation (8.2), and EGFR signaling (6.8). Cellular component categories (cytoplasm, nucleus) and molecular function terms (protein binding, receptor activity) were also significantly represented.

**Figure 12:**
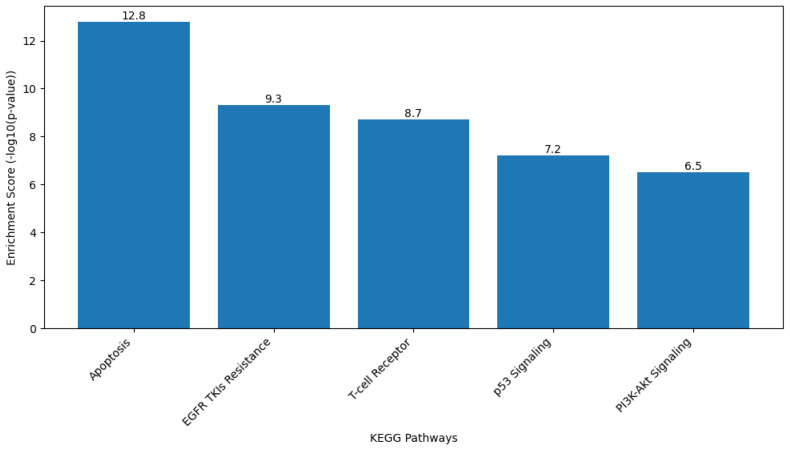
KEGG pathway enrichment analysis of the target genes CASP3, BAX, FOXP3, and EGFR. The analysis was performed using the DAVID Bioinformatics Resource (version 2024q1) and KEGG database, with statistical significance determined based on adjusted *p*-values (FDR < 0.05). The enrichment score is represented as −log10(*p*-value), where higher values indicate stronger pathway association. The most significantly enriched pathways included Apoptosis (Enrichment Score = 12.8), EGFR tyrosine kinase inhibitor resistance (9.3), and T cell receptor signaling (8.7). Additional pathways such as p53 signaling (7.2) and PI3K–Akt signaling (6.5) were also enriched.

Functional enrichment analysis was carried out to characterize the biological significance of the target genes CASP3, BAX, FOXP3, and EGFR using GO and KEGG databases. GO annotation categorized these genes into major functional groups, including biological processes, cellular components, and molecular functions. The results demonstrated significant enrichment in biological processes related to apoptotic regulation and immune signaling. Specifically, the genes were enriched in “apoptosis-induced proteolysis” (GO:0070059, FDR = 2.1 × 10^−8^), “regulation of T cell activation” (GO:0046630, FDR = 3.4 × 10^−6^), and “EGFR signaling pathway” (GO:0007173, FDR = 1.5 × 10^−5^) (Figure 11). KEGG pathway analysis further revealed that the target genes were significantly clustered in pathways directly associated with cancer pathogenesis and treatment response, including “Apoptosis” (hsa04210, FDR = 4.2 × 10^−7^), “EGFR tyrosine kinase inhibitor resistance” (hsa01521, FDR = 6.8 × 10^−6^), and “T cell receptor signaling pathway” (hsa04660, FDR = 1.1 × 10^−4^) (Figure 12). Overall, the combined GO and KEGG analyses showed that the studied genes were enriched in molecular mechanisms related to apoptosis regulation, immune modulation, and chemotherapeutic response. In addition, representative KEGG pathway maps corresponding to apoptosis (hsa04210), EGFR tyrosine kinase inhibitor resistance (hsa01521), and T-cell receptor signaling (hsa04660) have been included in the Supplementary Material as Figures S1–S3, respectively.

The enrichment analysis demonstrates that our target genes are functionally coordinated in key biological processes and pathways relevant to cancer pathogenesis, particularly in apoptosis, immune regulation, and growth factor signaling. This functional clustering provides mechanistic insights into how Hes and ADR might simultaneously target multiple interconnected pathways to exert their anticancer effects.

The GO and KEGG enrichment analyses were performed to gain a systems-level understanding of the biological significance of the experimentally validated target genes (CASP3, BAX, FOXP3, and EGFR). These bioinformatic approaches allowed the identification of enriched biological processes and signaling pathways that link apoptotic regulation, immune modulation, and cancer progression. Incorporating GO and KEGG analyses strengthened the study by integrating molecular and functional insights, thereby validating the experimental findings within broader biological networks.

#### 3.8.3. Promoter Region Analysis and Transcription Factor Binding Prediction

**Figure 13:**
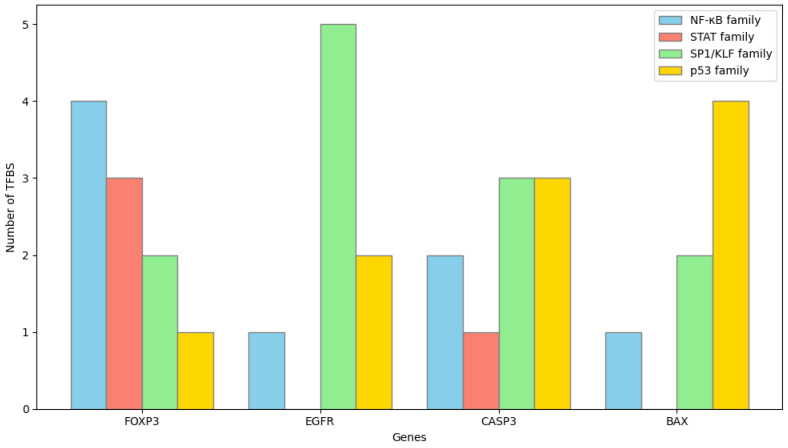
Distribution of predicted high-confidence TFBS across the promoter regions of the target genes CASP3, BAX, FOXP3, and EGFR. Promoter sequences spanning −1000 bp upstream to +500 bp downstream of TSS were analyzed using the JASPAR 2024 database. Only motifs with a relative profile score threshold > 85% were considered significant. Each bar represents the number of binding motifs identified for major transcription factor families (NF-κB, STAT, SP1/KLF, and p53). FOXP3 and EGFR promoters exhibited dense clustering of NF-κB and STAT motifs, whereas CASP3 and BAX promoters were enriched for p53-responsive and SP1 motifs.

**Figure 14:**
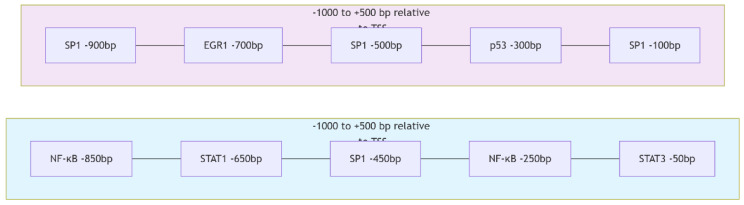
Promoter region architecture of FOXP3 and EGFR genes. The schematic depicts the relative positions of key predicted TFBS within −1000 bp to +500 bp of the transcription start site (TSS), as identified using JASPAR 2024 motif scanning (profile score > 85%). In the FOXP3 promoter (bottom panel), binding motifs for NF-κB (−850 bp, −250 bp), STAT1 (−650 bp), SP1 (−450 bp), and STAT3 (−50 bp) were detected. The EGFR promoter (top panel) contained multiple SP1 (−900 bp, −500 bp, −100 bp), EGR1 (−700 bp), and p53 (−300 bp) motifs.

Promoter region analysis was performed to identify transcription factors potentially regulating the expression of CASP3, BAX, FOXP3, and EGFR using the JASPAR 2024 database (https://jaspar.genereg.net). For each gene, promoter sequences corresponding to 1000 base pairs upstream and 500 base pairs downstream of the transcription start site (TSS) were retrieved from the UCSC Genome Browser based on the Homo sapiens GRCh38 reference genome. Motif scanning was conducted using position weight matrix models with a relative profile score threshold of 85% for significant transcription factor binding prediction. The analysis identified both common and gene-specific transcription factors associated with the regulation of these genes (Figure 13 and Figure 14). Multiple high-scoring motifs for NF-κB and STAT family transcription factors were detected within the FOXP3 promoter region. Binding sites for growth factor-related transcription factors such as SP1 and EGR1 were identified in the EGFR promoter.

The promoter architecture analysis revealed distinct regulatory landscapes for each target gene. The FOXP3 promoter was predominantly enriched with immune-responsive elements such as NF-κB and STAT family motifs, whereas the EGFR promoter displayed a higher density of growth factor- and stress-responsive motifs including SP1, EGR1, and p53. This gene-specific clustering pattern indicates that different upstream signaling cascades converge to modulate the transcriptional activity of these key oncogenic and immunoregulatory genes. The presence of multiple high-confidence TFBS within critical promoter regions provides mechanistic insight into how Hes and ADR combination therapy may exert coordinated transcriptional control, simultaneously attenuating proliferative (EGFR) and immunosuppressive (FOXP3) signaling axes in ovarian cancer cells.

#### 3.8.4. Structural Homology Modeling

**Figure 15:**
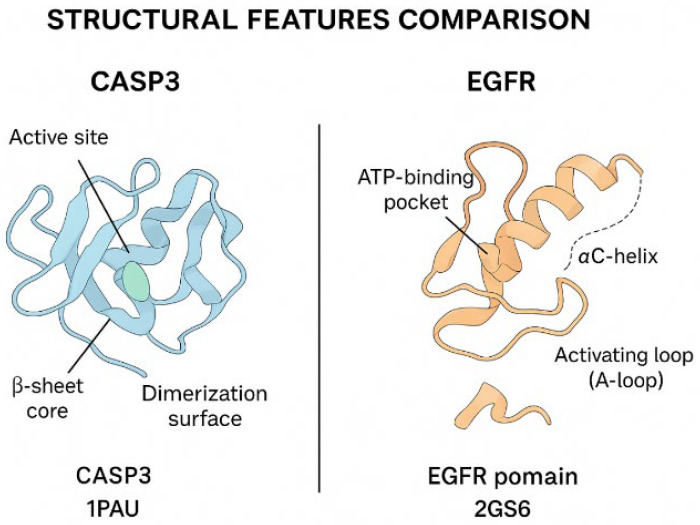
Structural features comparison of CASP3 and EGFR. Ribbon diagrams illustrate conserved motifs and domain organization within the modeled proteins. CASP3 (PDB template: 1PAU) shows a characteristic β-sheet core, catalytic active site, and dimerization surface typical of executioner caspases. EGFR (PDB template: 2GS6) highlights the ATP-binding pocket, αC-helix, and activation loop (A-loop) that are essential for tyrosine kinase regulation.

**Figure 16:**
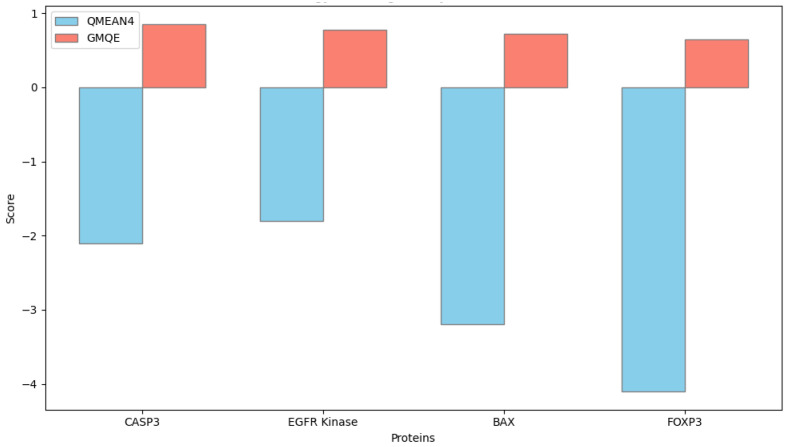
Quality assessment of homology models for target proteins. Comparative bar plot showing QMEAN4 and GMQE scores for CASP3, EGFR kinase, BAX, and FOXP3 models generated by SWISS-MODEL. QMEAN4 scores closer to zero indicate better agreement with high-resolution crystal structures, while GMQE values approaching 1.0 reflect higher model reliability. The obtained QMEAN4 (−1.8 to −4.0) and GMQE (0.6–0.8) scores demonstrate acceptable structural quality and consistency among the modeled proteins.

Homology modeling of CASP3 and EGFR proteins was performed using the SWISS-MODEL server to evaluate their structural characteristics within the apoptotic caspase and receptor tyrosine kinase families, respectively (Figure 15). The CASP3 model exhibited a canonical caspase-fold architecture, including the catalytic cysteine-histidine dyad within the active site (QMEAN = −2.1). The EGFR kinase domain model demonstrated a well-defined ATP-binding cleft and residues essential for receptor autophosphorylation and downstream signal transduction (QMEAN = −1.8). Comparative analysis confirmed structural consistency with experimentally resolved crystal templates (Figure 16).

In conclusion, the bioinformatics analyses confirmed that the four experimentally investigated target genes occupy central positions within interconnected apoptotic, proliferative, and immunological networks. These integrated findings provide a strong mechanistic basis suggesting that Hes and ADR exert a multifaceted anticancer effect through coordinated modulation of these key molecular pathways.

## 4. Discussion

This study provides a comprehensive evaluation of the antiproliferative, apoptotic, immunomodulatory, and antioxidant effects of Hes and ADR on SKOV3. The results demonstrated that Hes alone exhibits notable anticancer activity; however, this effect was markedly potentiated when combined with ADR. The combination treatment led to a pronounced reduction in cell viability after 48 h, confirming that Hes enhances the cytotoxic efficacy of ADR. This synergy, supported by the Chou–Talalay CI analysis, may be attributed to Hes-mediated cell cycle arrest and activation of apoptotic pathways. Similar synergistic behavior has been documented in various cancer cell lines where Hes enhanced the efficacy of chemotherapeutic agents [18,19,20].

At the molecular level, qRT-PCR analyses revealed that Caspase-3 and Bax expression increased significantly, with the combination group exhibiting the highest levels. These findings indicate that Hes + ADR robustly activates the intrinsic (mitochondrial) apoptotic pathway, consistent with previous studies reporting that Hes induces apoptosis in breast and cervical cancer cells by elevating the Bax/Bcl-2 ratio [20,21].

One of the most significant findings of this study is the suppression of EGFR and FOXP3 signaling. EGFR, a receptor tyrosine kinase involved in proliferation, metastasis, and drug resistance, was reduced by more than 50% following combination therapy, suggesting inhibition of proliferative signaling. FOXP3, a transcription factor linked to immunosuppression within the tumor microenvironment, also showed a marked decline in expression. Aberrant FOXP3 expression facilitates tumor immune evasion and supports oncogenic activation of pathways such as EGFR [22,23]. Therefore, simultaneous downregulation of EGFR and FOXP3 suggests that the combination therapy not only inhibits growth signaling but may also relieve immune suppression.

FOXP3 and EGFR were selected as representative molecular targets because they occupy central nodes in the network pharmacology and PPI analyses, linking proliferative and immunoregulatory signaling pathways. FOXP3 has been implicated in tumor-driven immune tolerance, whereas EGFR is a key oncogenic driver of proliferation and chemoresistance. GO and KEGG enrichment analyses demonstrated that both genes are functionally interconnected with apoptotic and cytokine-regulatory pathways, justifying their selection as primary targets for validation in this study. Although other oncogenic and tumor-suppressive genes were predicted in silico, FOXP3 and EGFR were prioritized for their dual mechanistic relevance and translational potential as therapeutic markers in ovarian cancer.

The cytokine data further support this immunomodulatory mechanism. While IL-6 and IFN-γ are generally categorized as pro-inflammatory cytokines, their roles are highly context-dependent. In the tumor microenvironment, transient or moderate upregulation of these cytokines can contribute to immunogenic activation rather than chronic inflammation. IFN-γ promotes cytotoxic T-cell and NK-cell activity, enhancing antitumor immunity, whereas IL-6 supports differentiation of effector T cells and tissue repair when maintained within physiological limits. In the present study, the concurrent elevation of IL-6 and IFN-γ with suppression of TNF-α suggests a controlled immunoregulatory response rather than an uncontrolled inflammatory cascade. Therefore, the cytokine pattern induced by Hes treatment may represent a balanced immune activation state favoring antitumor defense while minimizing excessive inflammation. Hes treatment alone elevated IL-6 and IFN-γ while reducing TNF-α, indicative of an anti-inflammatory and immune-stimulating effect. Conversely, ADR alone suppressed IL-6 and IFN-γ while increasing TNF-α, a hallmark of chemotherapy-induced inflammatory stress. In the combination group, decreased TNF-α and increased IFN-γ levels indicate that Hes compensates for ADR-induced inflammation, shifting the microenvironment toward an immune-stimulatory state [19,24].

Enhanced TAC observed in Hes-treated groups further highlights the redox-modulating potential of Hes. By scavenging ROS, Hes reduces oxidative stress and preserves cellular integrity. This property likely contributes to its protective role against ADR-induced oxidative toxicity. Previous studies have confirmed that Hes elevates the activities of endogenous antioxidant enzymes such as SOD, CAT, and GPx, decreases lipid peroxidation (MDA levels), and maintains mitochondrial function [25,26,27,28]. Consequently, the Hes + ADR combination achieves dual benefits: potentiating cytotoxicity in tumor cells while mitigating oxidative damage in normal cells.

In a broader mechanistic context, integration of apoptotic, antioxidant, and immunological data reveals that Hes enhances ADR efficacy through both redox and immune modulation. The combined treatment not only induced intrinsic apoptosis (as indicated by Caspase-3 and Bax upregulation) but also remodeled the cytokine milieu toward an immunogenic phenotype, reminiscent of immunogenic cell death. Consistent with our previous findings in HeLa cells [19], Hes co-administration significantly downregulated IL-6 and TNF-α while increasing IFN-γ secretion. This cytokine pattern suggests a transition from chronic inflammation to a cytotoxic immune response, characteristic of ICD [29].

ICD is a regulated form of apoptosis associated with the release of damage-associated molecular patterns (DAMPs), including calreticulin, HSP70/90, HMGB1, and prothymosin-α, which activate dendritic and cytotoxic T cells [10]. Rodrigues et al. demonstrated that anthracyclines such as doxorubicin induce ICD through DAMP exposure and immune activation [29]. Jiang et al. further showed that mitochondrial oxidative stress and ROS-ER stress crosstalk link ICD to redox signaling [30]. In the present study, the controlled increase in TAC following Hes + ADR treatment suggests that Hes moderates oxidative stress to an optimal level—sufficient to trigger apoptosis and ICD without inducing necrosis.

Lucarini et al. previously reported that anthracyclines are potent ICD inducers, upregulating MHC-I and IFN-γ expression in CD8^+^ T cells [31]. Our data, showing strong IFN-γ induction in the combination group, align with this concept and support Hes-mediated enhancement of ADR-induced ICD through redox-dependent sensitization. Reviews by Rahmani et al. and Aggarwal et al. [32,33] have established that Hes modulates ERK/MAPK, STAT3, and NF-κB signaling, activates p53, and enhances mitochondrial apoptosis, mechanisms that may collectively explain the suppression of IL-6 and TNF-α and activation of apoptotic genes observed here.

Franzese et al. highlighted that apoptosis and immune activation are metabolically coupled, with optimal therapy achieved when tumor apoptosis coexists with preserved T-cell metabolic activity [34]. In our study, the concurrent downregulation of immunosuppressive cytokines (IL-6, TNF-α) and elevation of IFN-γ likely support this paradigm by reducing T-cell exhaustion and promoting durable antitumor immunity.

These experimental findings are consistent with clinical evidence. Buzdağlı et al. showed that Hes supplementation significantly lowers circulating CRP, IL-6, and TNF-α, while enhancing systemic antioxidant capacity (TAC, SOD, GSH) [35]. Similarly, Lee et al. demonstrated that Hes and its lipophilic derivatives suppress UV-induced IL-6 and TNF-α expression and exhibit potent antioxidant activity [36]. Together, these data underscore that Hes exerts a structurally dependent, dose-sensitive antioxidant and anti-inflammatory effect that complements chemotherapy.

In the context of ICD biomarker discovery, Birmpilis et al. identified DAMPs such as prothymosin-α (proTα) as correlates of apoptosis intensity and indicators of immunogenicity [37]. Given that the Hes + ADR group exhibited the highest apoptotic ratio (~59%), similar DAMP-mediated immune activation may underlie the observed IFN-γ upregulation.

Collectively, these results support a multifactorial model in which Hes acts as a biochemical synchronizer that aligns redox homeostasis, apoptosis, and immune activation. By constraining excessive ROS generation while maintaining apoptotic thresholds necessary for DAMP release, Hes transforms ADR-induced cytotoxicity into a coordinated apoptotic–immunogenic process. This mechanistic integration, supported by molecular, metabolic, and bioinformatic evidence, underscores the translational potential of Hes + ADR combination therapy in overcoming chemoresistance and eliciting a durable antitumor immune response in ovarian cancer.

Recent in vivo studies further substantiate the translational potential of combining Hes with doxorubicin. Patel and Shah [38] demonstrated that Hes pretreatment (200 mg/kg) markedly reduced tumor incidence, oxidative stress, and inflammatory cytokine levels in a DMBA-induced breast cancer rat model, while simultaneously mitigating doxorubicin-related systemic toxicity. Shakiba et al. reported consistent findings in a 4T1 murine breast cancer model, where co-administration of Hes (20 mg/kg) and doxorubicin (10 mg/kg) yielded an 80% survival rate, elevated IFN-γ levels, and suppression of pro-angiogenic and proliferative markers (VEGFa, MMP9, Ki-67). These results mirror the cytokine and apoptotic gene responses observed in the present in vitro SKOV3 study, reinforcing that Hes + ADR combination triggers apoptosis and immunogenic remodeling across models [39].

From a pharmacokinetic and formulation standpoint, the bioavailability of Hes remains a limiting factor for clinical translation. As summarized by Wdowiak et al. [40], Hes exhibits poor aqueous solubility and low intestinal absorption, which restrict systemic exposure; however, encapsulation in nanoparticles or liposomal carriers and co-administration with probiotics significantly enhances its oral bioavailability. These strategies are highly relevant to optimizing systemic delivery in animal or clinical settings and align with our current proposal for nanoparticle-based formulation development.

Furthermore, the cardioprotective potential of Hes is critical when considering doxorubicin’s dose-dependent cardiotoxicity. A recent study in Wistar rats showed that Hes (50 mg/kg) co-administration normalized ECG parameters, blood pressure, and serum pro-BNP levels, confirming its ability to protect against doxorubicin-induced cardiac injury. This evidence collectively underscores that the Hes + ADR combination may achieve in vivo synergy not only through enhanced tumor suppression but also via systemic protection mechanisms, bridging the gap between cellular efficacy and physiological safety [41].

Although this study provides compelling experimental and bioinformatic evidence for the synergistic anticancer effects of Hes and ADR in ovarian cancer cells, several limitations should be acknowledged. First, the work was conducted in a single in vitro model (SKOV3), which may not fully recapitulate the complex tumor microenvironment of ovarian carcinoma. It should also be noted that the SKOV3 ovarian cancer cell line used in this study is TP53-null, lacking functional p53 protein expression. Therefore, the apoptotic and cytotoxic responses observed are likely mediated through p53-independent mechanisms. The absence of p53 function provides a unique model for investigating alternative apoptotic pathways activated by Hes and ADR, including those involving mitochondrial and redox regulation. However, this also represents a biological limitation, as cells with functional TP53 may exhibit distinct sensitivity patterns. Future studies using additional ovarian cancer cell lines with different TP53 backgrounds—such as A2780 (wild-type TP53) or OVCAR3 (mutant TP53)—will be essential to determine whether p53 signaling modulates the synergistic cytotoxic and apoptotic effects observed here. Second, while gene expression analyses provided mechanistic insight into apoptosis, oxidative balance, and cytokine modulation, protein-level confirmation (e.g., via Western blot or immunocytochemistry) was not performed. Third, the study did not include flow cytometric quantification of apoptosis or in vivo pharmacokinetic evaluation, which are essential to confirm therapeutic synergy and bioavailability. Moreover, the potential cardioprotective effects of Hes against ADR-induced toxicity—a clinically relevant aspect—remain to be validated in animal models. Although non-cancerous ovarian epithelial cell lines (e.g., IOSE-80 or HOSE) were not included in this study, our primary objective was to elucidate molecular mechanisms of synergistic cytotoxicity in SKOV3 malignant cells. Future investigations will incorporate parallel assays in non-cancerous epithelial models to assess selective cytotoxicity and therapeutic specificity. Therefore, although the findings establish a robust molecular basis for synergy, translational conclusions should be drawn cautiously until confirmed by preclinical and clinical studies.

Future research should aim to validate these findings in multiple ovarian cancer cell lines and animal models to better understand the pharmacodynamic and pharmacokinetic interactions between Hes and ADR. Future investigations should also evaluate the selectivity of the Hes and ADR combination between normal ovarian epithelial and malignant cells to clarify therapeutic specificity and potential off-target toxicity. Moreover, comparative cytotoxicity studies in non-malignant ovarian epithelial cells will be essential to determine the therapeutic window and validate the selective anticancer efficacy of the Hes and ADR combination. Furthermore, studies incorporating three-dimensional culture or tumor microenvironment models would help assess immunologic and stromal interactions relevant to translational applications. Integrative in vivo studies evaluating systemic oxidative stress, immune cell infiltration, and cardiotoxicity biomarkers would provide critical translational data. Advanced omics-based approaches—such as transcriptomic, proteomic, and metabolomic profiling—could further elucidate how this combination modulates global cellular networks. Moreover, molecular docking and dynamics simulations using the modeled CASP3 and EGFR structures from this study could identify potential binding sites and predict ligand interactions. Clinical translation will also benefit from formulation research to enhance Hes’s bioavailability, including nanoparticle encapsulation or liposomal delivery systems. Ultimately, combining natural flavonoids with standard chemotherapeutics represents a promising strategy for developing redox- and immunity-targeted combination therapies capable of overcoming chemoresistance and minimizing systemic toxicity in ovarian cancer.

Overall, the present findings indicate that Hes, when used as an adjuvant to ADR, enhances therapeutic efficacy through complementary molecular and functional mechanisms. The combined treatment reduced cell viability (MTT assay), activated intrinsic apoptosis via Caspase-3 and Bax upregulation (qRT-PCR), and suppressed proliferative and immunosuppressive signaling by downregulating EGFR and FOXP3. ELISA analyses confirmed modulation of the cytokine profile, characterized by decreased TNF-α and increased IFN-γ, while antioxidant assays demonstrated significant restoration of redox balance through elevated TAC. Bioinformatic validation, including PPI network, GO/KEGG enrichment, and promoter region analyses, supported these experimental findings by linking the four target genes to convergent apoptotic, proliferative, and immunoregulatory pathways. Collectively, these integrated data demonstrate that Hes not only potentiates ADR’s anticancer activity but also mitigates its oxidative and inflammatory toxicity, suggesting that this natural compound may serve as a promising adjunctive candidate in combination chemotherapy for ovarian cancer.

## 5. Conclusions

This study systematically investigated the cytotoxic, apoptotic, immunomodulatory, and antioxidant effects of Hes and ADR in SKOV3 ovarian cancer cells through a combination of experimental and bioinformatic approaches. MTT assays demonstrated a time- and dose-dependent decrease in cell viability, with the Hes + ADR combination producing a marked synergistic cytotoxic effect. Gene expression analysis confirmed activation of intrinsic apoptosis via upregulation of Caspase-3 and Bax, while FOXP3 and EGFR expression levels were significantly reduced, indicating suppression of immunosuppressive and proliferative signaling pathways. ELISA measurements revealed that Hes modulated cytokine profiles by decreasing TNF-α and enhancing IFN-γ, suggesting restoration of immune balance. In parallel, spectrophotometric assessment of TAC showed that Hes effectively counteracted ADR-induced oxidative stress. Bioinformatic analyses, including PPI network mapping, GO/KEGG enrichment, and promoter-region exploration, supported these experimental results by demonstrating that the target genes are functionally interconnected within apoptotic, proliferative, and immunoregulatory networks.

**Figure 17:**
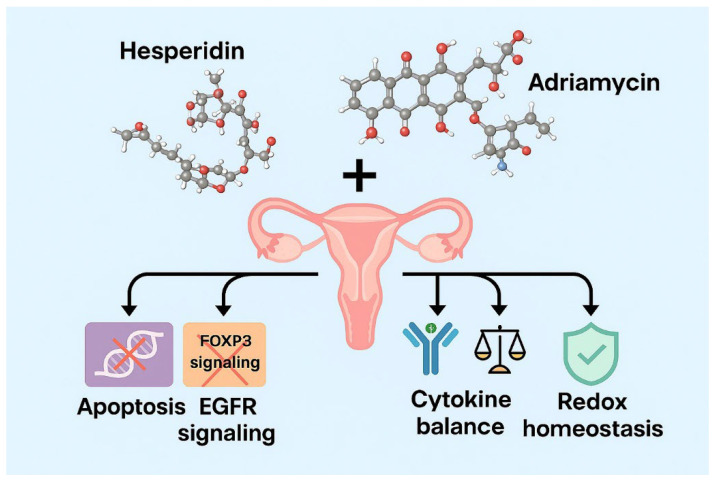
Proposed schematic summary illustrating the synergistic anticancer mechanism of Hes and ADR in SKOV3 ovarian cancer cells. The combination treatment coordinately enhances mitochondrial apoptosis (↑ Bax, ↑ Caspase-3), suppresses proliferative (EGFR) and immunosuppressive (FOXP3) signaling, restores cytokine homeostasis (↓ TNF-α, ↑ IFN-γ), and maintains redox equilibrium by modulating ROS levels. The overall network interaction highlights the integrated pro-apoptotic, anti-inflammatory, and antioxidant actions of the Hes + ADR combination, providing a mechanistic framework for its synergistic antitumor efficacy.

Collectively, these integrated data demonstrate that the combination of Hes with ADR exerts multifaceted anticancer effects at the cellular level by coordinating apoptosis induction, inhibition of FOXP3 and EGFR signaling, regulation of cytokine homeostasis, and preservation of redox balance. As summarized in the proposed graphical model (Figure 17), these interactions illustrate how Hes synergistically enhances ADR efficacy through convergent regulation of apoptotic, immunologic, and redox pathways. These findings are confined to bench-side evidence and provide an experimental foundation for understanding the mechanistic interactions between Hes and ADR in vitro.

## Figures and Tables

**Table 1:** Primer sequences used in qRT-PCR.

Gene	Primer Sequence (5′ → 3′)	Product Size (bp)
Caspase-3	F: AGAGGGGATCGTTGTAGAAGCTG R: CACAAGCGACTGGATGAACCA	120
Bax	F: TCTGACGGCAAACTTGCACT R: CAAAGTAGAAAAGGGCGACAAC	145
FOXP3	F: GAAACAGCACATTCCCAGAGTTC R: ATGGCCCAGCGGATGAG	138
EGFR	F: CCTCACAGCAGGGTCTTCTC R: TGGCTCACCCTCCAGAAGTT	112
GAPDH	F: GGAGCGAGATCCCTCCAAAAT R: GGCTGTTGTCATACTTCTCATGG	197

## Data Availability

All experimental data generated or analyzed during this study are available from the corresponding author upon reasonable request. The bioinformatics data supporting the findings of this study were obtained from publicly accessible databases (STRING, DAVID, UCSC Genome Browser, JASPAR, and SWISS-MODEL), as described in the Section 2.

## Supplementary Materials

The following supporting information can be downloaded at: https://www.mdpi.com/article/10.3390/biomedicines13112798/s1, Figure S1. KEGG Apoptosis Pathway (hsa04210). This figure presents the KEGG apoptosis signaling pathway, illustrating the extrinsic, intrinsic (mitochondrial), and ER-stress–mediated apoptotic cascades. Key nodes such as CASP3, BAX, and associated regulators involved in apoptotic signaling are shown to provide functional context for the genes examined in this study. Figure S2. KEGG EGFR Tyrosine Kinase Inhibitor Resistance Pathway (hsa01521). This figure depicts the EGFR tyrosine kinase inhibitor resistance pathway, highlighting upstream activators, bypass signaling mechanisms, and downstream molecular routes—such as PI3K–Akt and MAPK pathways—relevant to EGFR-mediated proliferation and drug resistance mechanisms. Figure S3. KEGG T-Cell Receptor Signaling Pathway (hsa04660). This figure illustrates the T-cell receptor signaling cascade, showing proximal and distal signaling molecules, transcription factors, and cytokine outputs. The pathway provides immunological context for understanding FOXP3- and cytokine-related regulatory interactions discussed in the study.

## Abbreviations

The following abbreviations are used in this manuscript:

ADR	Adriamycin
Hes	Hesperidin
SKOV3	Human ovarian adenocarcinoma cells
FOXP3	Forkhead box P3
EGFR	Epidermal growth factor receptor
ROS	Reactive oxygen species
MTT	3-(4,5-dimethylthiazol-2-yl)-2,5-diphenyltetrazolium bromide
IC_50_	Half maximal inhibitory concentration
CI	Combination Index
Fa	Fraction Affected
IL-6	Interleukin-6
IFN-γ	Interferon-gamma
TNF-α	Tumor necrosis factor-alpha
TAC	Total antioxidant capacity
qRT-PCR	Quantitative real-time polymerase chain reaction
cDNA	Complementary DNA
Ethylenediaminetetraacetic acid	EDTA
Fluorescein isothiocyanate	FITC
GO	Gene Ontology
KEGG	Kyoto Encyclopedia of Genes and Genomes
DAVID	Database for Annotation, Visualization, and Integrated Discovery
TFBS	Transcription factor binding sites
PDB	Protein Data Bank
GMQE	Global Model Quality Estimation
Roswell Park Memorial Institute (RPMI)-1640 medium	RPMI-1640
