# Supplementary figures and images for: Integrated Experimental and Bioinformatic Analysis Reveals Synergistic Apoptotic, Antioxidant, and Immunomodulatory Effects of Hesperidin and Adriamycin in SKOV3 Ovarian Cancer Cells
Source: Biomedicines. 2025 Nov 17;13(11):2798. doi: 10.3390/biomedicines13112798 (PMC12650299; doi:10.3390/biomedicines13112798)

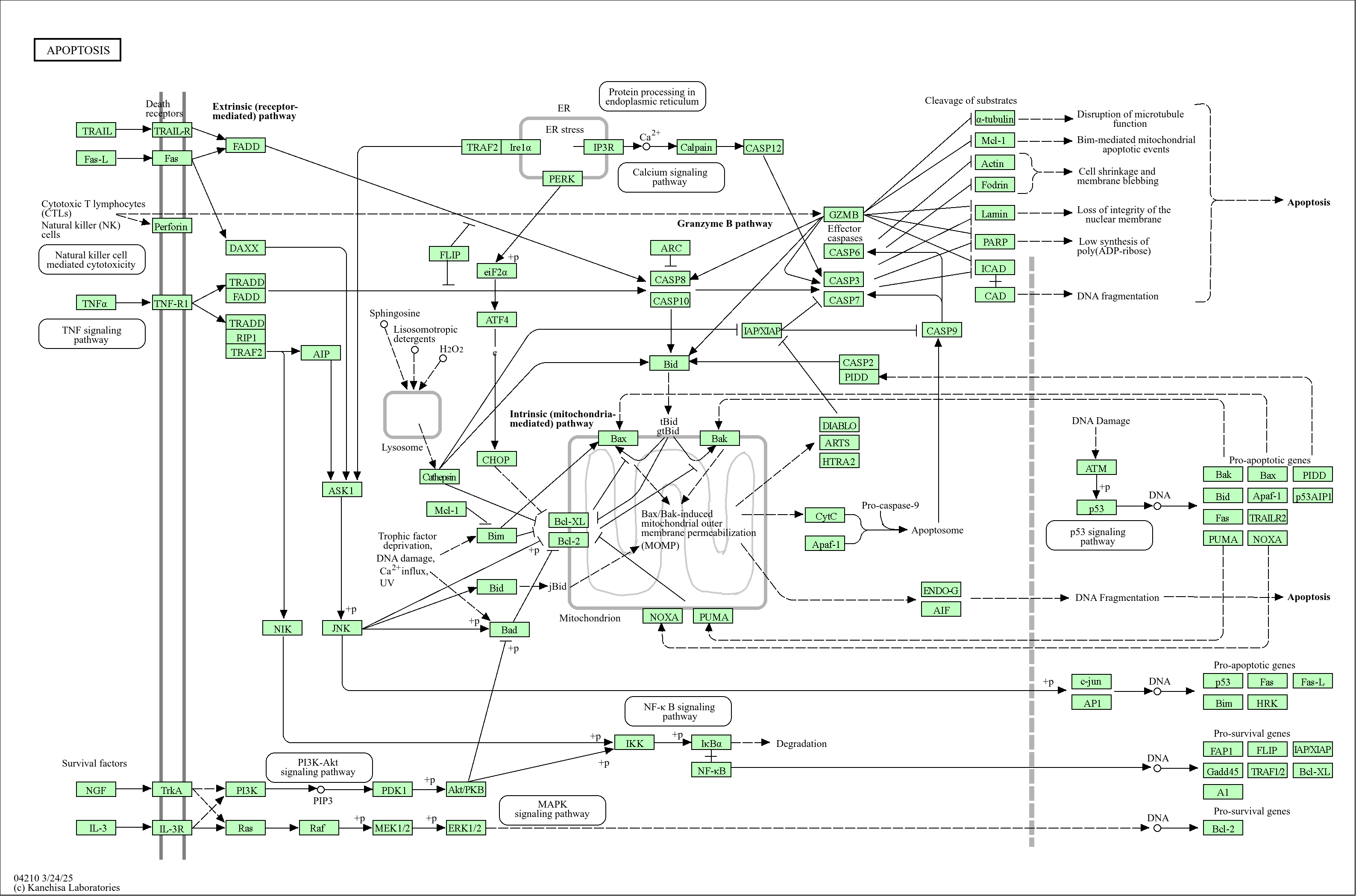

Supplement: Supplementary file 1 [file biomedicines-13-02798-s001.zip › Figure S1.jpg]

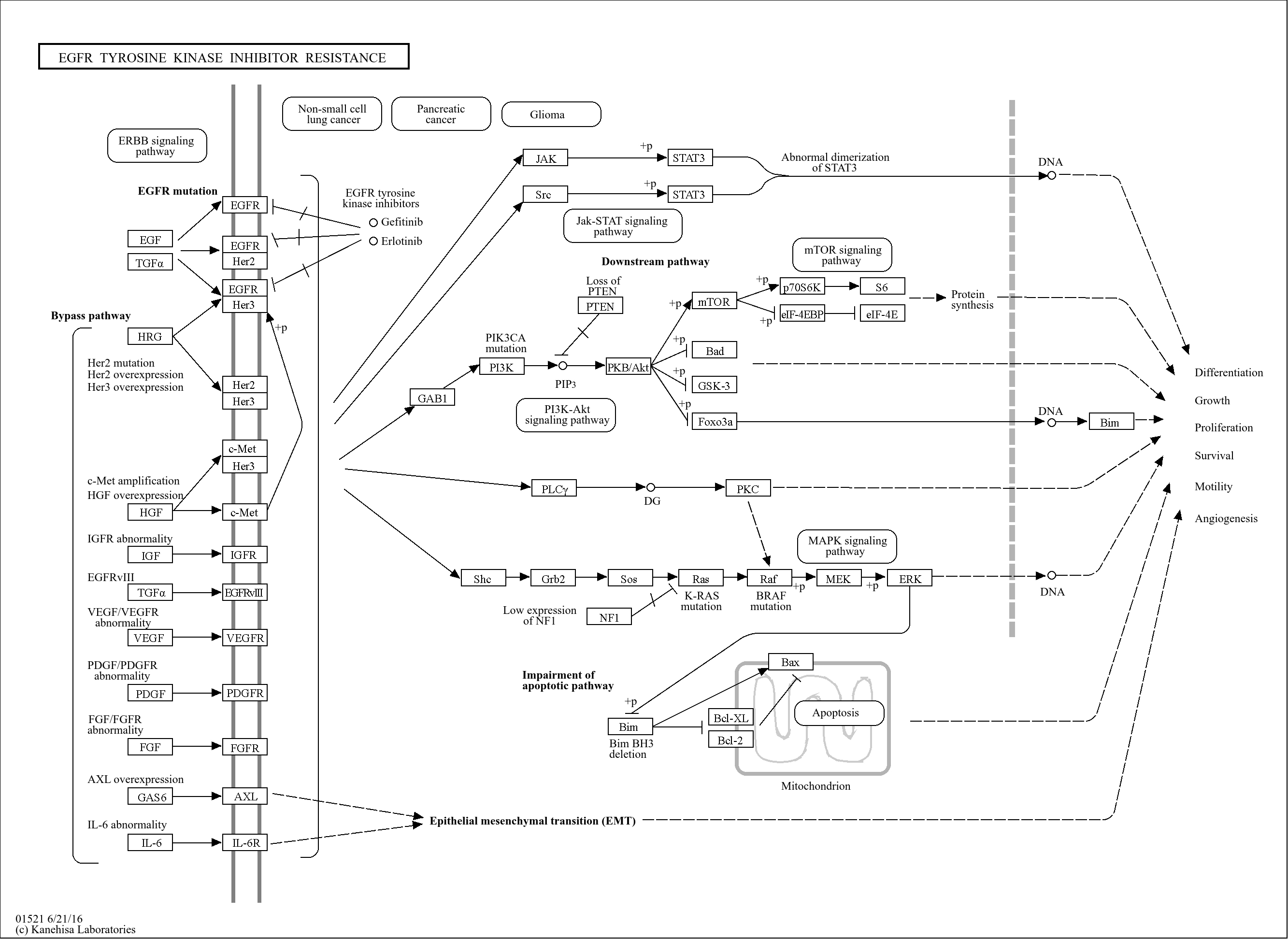

Supplement: Supplementary file 1 [file biomedicines-13-02798-s001.zip › Figure S2.jpg]

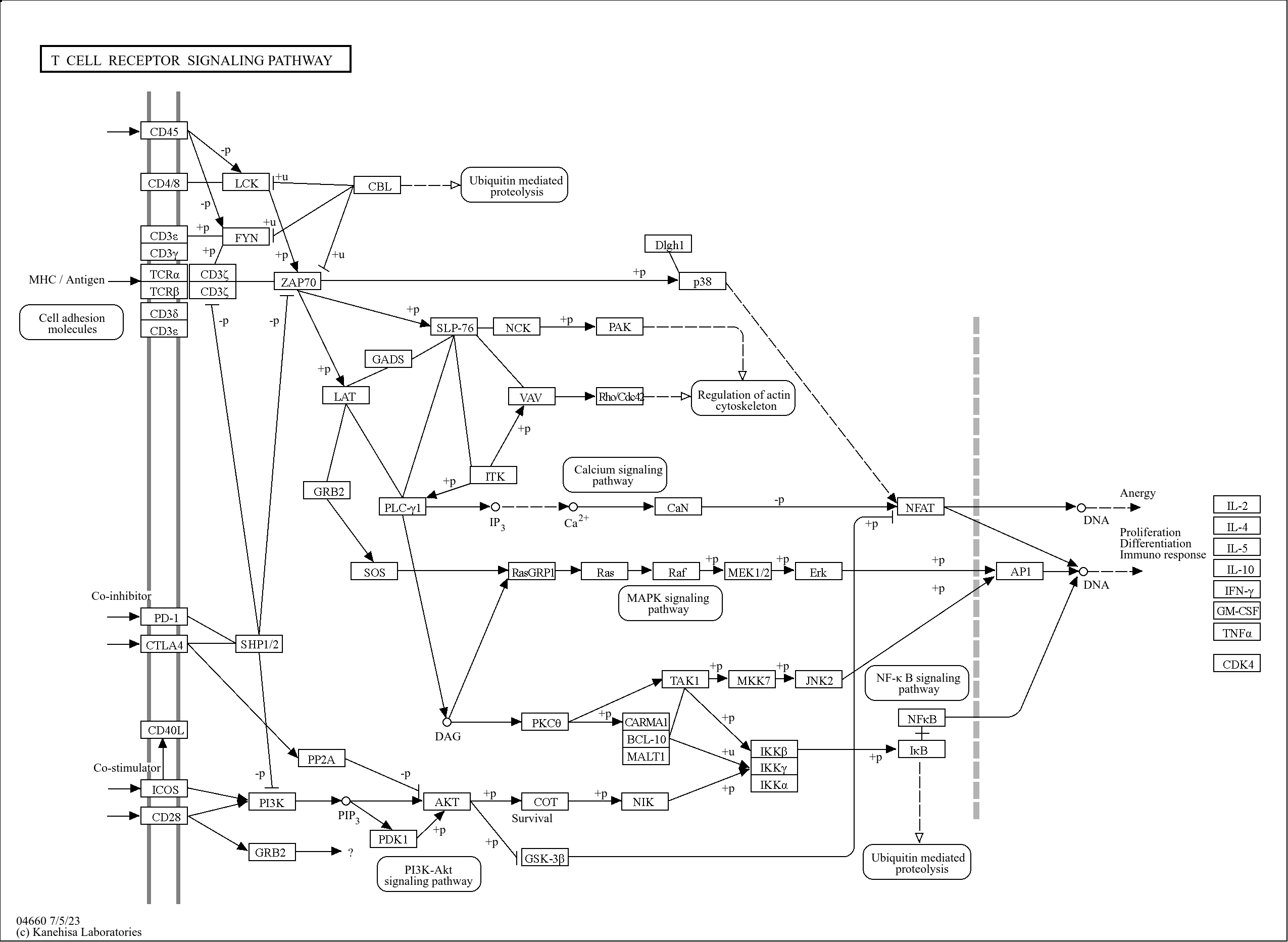

Supplement: Supplementary file 1 [file biomedicines-13-02798-s001.zip › Figure S3.jpg]
